# Study on the Quality Change and Regulation Mechanism of ‘Shannongsu’ Pear Under Low-Temperature Storage

**DOI:** 10.3390/ijms26072900

**Published:** 2025-03-22

**Authors:** Cong Chen, Sumin Qi, Susu Zhang, Ruize Hu, Lu Li, Xinyue Zhou, Nan Wang, Xuesen Chen, Zongying Zhang

**Affiliations:** 1 College of Horticultural Science and Engineering, Shandong Agricultural University, Tai’an 271018, China; 15006564462@163.com (C.C.); zhangss0116@163.com (S.Z.); 13105385516@163.com (R.H.); 18262163126@163.com (L.L.); 15269676816@163.com (X.Z.); nanwangjingzi@163.com (N.W.); chenxs@sdau.edu.cn (X.C.); 2 Shandong Institute of Pomology, Tai’an 271000, China; qsm20095089@126.com

**Keywords:** Shannongsu pear, low temperature, ethylene, texture, saccharic acid, aroma, ascorbic acid content

## Abstract

‘Shannongsu’ pear is a new high-quality cultivar. To ascertain the storage characteristics of ‘Shannongsu’ pears at low temperatures (0 ± 0.5 °C), the following parameters were determined: fruit firmness, ethylene, aromatic compounds, sugar content, acidity, ascorbic acid, and the expression levels of ethylene-related genes and texture-softening genes. The firmness of ‘Shannongsu’ pears changed less than that of the control, decreasing by only 18.8% after 170 days of storage. Low temperatures suppressed the expression of key genes associated with *PbACS1a* and *PbACO1*. Moreover, the expression of key genes related to fruit softening (*PbPG1*, *PbXET*, *PbPME*, and *Pbα-L-Af*) was suppressed during storage at low temperatures and remained at low levels. Therefore, the low levels of ethylene biosynthesis and the expression of key genes involved in fruit softening might play a major role in the excellent storage characteristics of the ‘Shannongsu’ cultivar. After 170 days of storage, ‘Shannongsu’ pears did not show significant changes in key quality dimensions such as firmness, sugar, acid, sugar–acid ratio, and ascorbic acid content. Therefore, low temperatures could help maintain the freshness, flavor, and nutritional quality of the ‘Shannongsu’ pear. Our findings reveal for the first time the low-temperature storage characteristics of ‘Shannongsu’ pears, providing a new scientific theoretical basis for pear production and marketing.

## 1. Introduction

Pears are popular among consumers worldwide and are the second-largest fruits produced by deciduous fruit trees in China, enabling increases in fruit farmers’ income and rural revitalization. However, with the continuous development of the national economy, the main cultivated pear varieties in China, such as the ‘Dangshansu’ pear and the ‘Yali’ pear, are characterized by being late-maturing but of inferior quality, having delicate flavors and a high stone cell content. These characteristics constrain the profit margins of both fruit farmers and dealers, preventing them from meeting the demands of high-end consumers. Consequently, there has been an overall continuous downturn in China’s pear consumption market, accompanied by an overcapacity phenomenon [1]. To meet the huge market needs of China’s population of 1.4 billion and promote the efficient development of China’s pear industry, varieties integrating high quality, storability, and late maturity are essential. Therefore, in 2003, a crossbreeding program was initiated using the ‘Xinli 7’ pear—which has a complex genetic background—as the female parent and the ‘Dangshansu’ pear, a late-maturing and storage-resistant maincrop variety, as the male parent. After ten years of research, a new high-quality pear variety, ‘Shannongsu’—which matures at a very late stage—passed the validation criteria of the Shandong Provincial Crop Variety Validation Committee in 2015. The ‘Shannongsu’ pear has outstanding advantages, including large fruit size, fine and crispy flesh, a high sugar content, and resistance to browning. It has become a preferred variety among pear cultivars [2].

The storage quality of fruits, crucial for their potential as leading cultivars and commodities, has drawn extensive academic research focusing on the mechanisms of its formation and maintenance [3]. It has been found that changes in fruit texture affect the freshness and quality and are also crucial for determining storage quality, which is regulated by various factors. Among these, the hormone ethylene plays an important regulatory role [4,5]. Exogenous ethylene treatment expedites fruit softening by increasing the endogenous ethylene concentration in fruits [6]. 1-Methylcyclopropene (1-MCP), an inhibitor of the ethylene response, also can effectively retard the fruit softening process and extend the shelf life of fruits [7,8]. Silencing the expression of 1-aminocyclopropane-1-carboxylate synthase (ACS) and 1-aminocyclopropane-1-carboxylate oxidase (ACO)—two key genes for ethylene biosynthesis—can significantly reduce endogenous ethylene synthesis in fruits, delaying fruit softening and prolonging fruit storage [9]. The process of fruit texture softening is very complex, and many studies have shown that it is closely related to changes in the cell wall structure and its components [10]. During the storage of apples, components such as pectin and cellulose in the cell wall are gradually hydrolyzed due to interactions with enzymes such as α-L-arabinofuranosidase (α-L-Af), β-galactosidase (β-Gal), polygalacturonase (PG), xyloglucan endoglycosyltransferase (XET), and pectin methyl esterase (PME), resulting in a decrease in firmness. Previous studies have shown that cell wall enzymes play an important role in fruit softening. Both a temperature of 0 °C and inhibitors such as 1-MCP can inhibit the enzyme activity and delay the decline in firmness [11].

The fruit aroma not only gives a specific flavor but is also one of the critical factors affecting fruit storage quality. The expression of key genes involved in the fruit aroma synthesis pathway is regulated by ethylene. When ethylene from fruit is inhibited, the levels of aroma components are also changed [12]. In addition, factors such as the sugar, acid, and ascorbic acid contents are important indicators of fruit flavor and nutritional quality. Studies show that the content of organic acids in non-storable common tomatoes decreased sharply during storage, while that in storable bunch tomatoes decreased more slowly [13]. Therefore, storage-tolerant varieties that exhibit improved texture, flavor, and nutritional quality over a longer duration are the most economical and effective way to achieve a stable annual supply of bulk fruits.

Although extensive research has been carried out on the formation and maintenance of fruit storage quality, the mechanism underlying fruit quality maintenance remains unclear due to significant varietal differences. Findings indicate that the ‘Shannongsu’ pear maintains stable firmness and other quality-related traits during 60 days of room-temperature storage, demonstrating good storage resistance. However, its storage characteristics at low temperatures are yet to be elucidated [14].

Therefore, in this study, the ‘Shannongsu’ pear was used as the test material, while ‘Dangshansu’ and ‘Qiuyue’ pears served as controls. The ‘Dangshansu’ pear, the parent and main cultivated variety of the ‘Shannongsu’ pear, is closely related to the variety characteristics of the latter. The ‘Qiuyue’ pear, with a wide planting area and good economic performance in current agricultural production, was also selected. Therefore, ‘Dangshansu’ and ‘Qiuyue’ pears were selected as controls, rendering this study highly representative and valuable [15]. By measuring and analyzing indicators such as firmness and ethylene, we can elucidate the variation rules of the nutritional quality of ‘Shannongsu’ pears during low-temperature storage and the corresponding regulatory mechanisms. Comparisons among ‘Shannongsu’, ‘Dangshansu’, and ‘Qiuyue’ pears will identify the similarities and differences in nutritional quality changes among the three pear varieties during low-temperature storage. This will further enrich our understanding of the formation mechanisms of storage quality in different pear varieties, providing a scientific basis for the genetic breeding, improvement, and storage of new varieties.

## 2. Results

### 2.1. Changes in Firmness and Ethylene During Low-Temperature Storage

**Figure 1:**
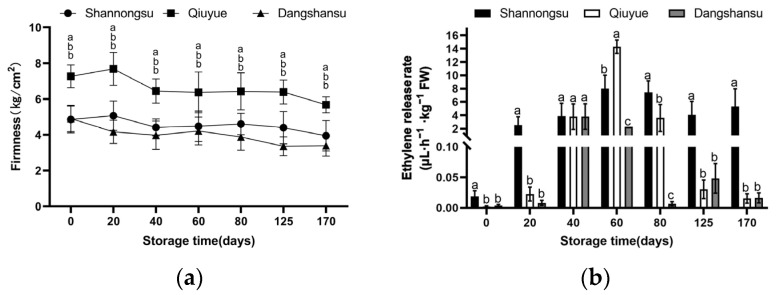
Changes in firmness and ethylene release rate during storage: (**a**) firmness; (**b**) ethylene release rate. Values shown represent means ± SD (*n* = 3). Different letters indicate significant differences among varieties at the 0.05 level.

As shown in Figure 1a, the firmness of ‘Shannongsu’ and ‘Dangshansu’ pears was comparable 0 days after harvesting, and both were lower than that of the ‘Qiuyue’ pear. After 170 d of low-temperature storage, the firmness of the ‘Dangshansu’ pear decreased by 30.9% from 4.9 kg/cm^2^ to 3.38 kg/cm^2^. The firmness of the ‘Shannongsu’ pear decreased by only 18.8%, reaching 3.94 kg/cm^2^, maintaining the fruit’s crispness and quality. The firmness of the ‘Qiuyue’ pear decreased by 21.8%, dropping from 7.26 kg/cm^2^ to 5.68 kg/cm^2^. After 125 d of storage, ‘Qiuyue’ pears exhibited skin breakage and rotting, while ‘Shannongsu’ and ‘Dangshansu’ pears did not exhibit this phenomenon.

Figure 1b shows that low temperatures are more effective in reducing the increase in ethylene production of pear fruits of the three varieties during storage. The ethylene of the ‘Shannongsu’ pear during storage shows a small amplitude, while that of the ‘Dangshansu’ and ‘Qiuyue’ pears shows a trend of first increasing and then decreasing. After 40 days of storage, ‘Dangshansu’ pears reached their peak ethylene release earliest, with the maximum ethylene release being 3.8 (μL h^−1^ ·kg^−1^ FW). When stored for 60 days, the ethylene production of ‘Shannongsu’ pears reached a maximum value of only 8 (μL h^−1^·kg^−1^ FW), which was between that of ‘Dangshansu’ pears (2.3 μL h^−1^·kg^−1^ FW) and ‘Qiuyue’ pears (14.3 μL h^−1^·kg^−1^ FW).

### 2.2. Changes in Sugar and Acid Contents During Low-Temperature Storage

**Figure 2:**
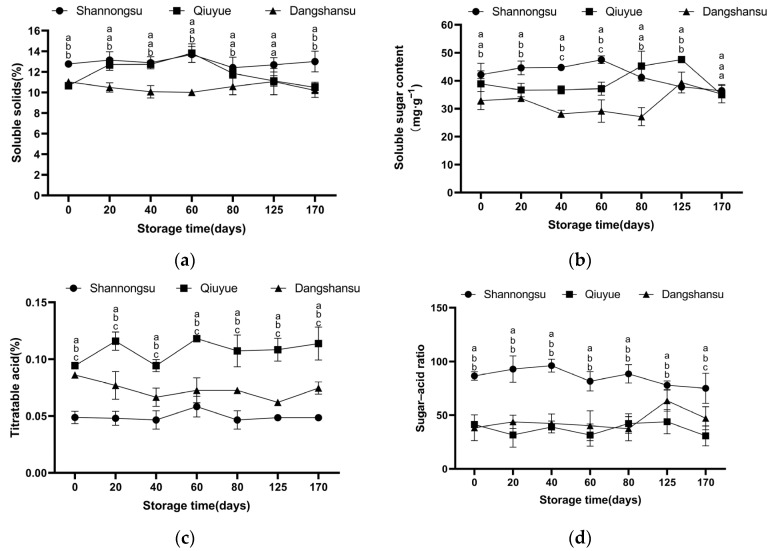
Changes in soluble solids, soluble sugars, titratable acid content, and sugar–acid ratio during storage: (**a**) soluble solids; (**b**) soluble sugar content; (**c**) titratable acid content; (**d**) sugar–acid ratio. Values shown represent means ± SD (*n* = 3). Different letters indicate significant differences among varieties at the 0.05 level.

Figure 2a–d show that the low temperature more effectively slowed down the decrease in the contents of soluble sugar and titratable acid in the fruits of the three varieties. During storage, the titratable acidity content of the ‘Shannongsu’ pear was lower than that of the ‘Dangshansu’ and ‘Qiuyue’ pears, while its total soluble sugar content was generally higher. Moreover, the sugar–acid ratio of the ‘Shannongsu’ pear was significantly higher than that of the two control varieties (*p* < 0.05): it reached 86.49 on day 0 after harvest, far higher than that of the ‘Dangshansu’ pear (38) and the ‘Qiuyue’ pear (41). After 170 days of storage, the sugar–acid ratio of the ‘Shannongsu’ pear decreased to 74.99 but was still higher than that of the ‘Dangshansu’ and ‘Qiuyue’ pears.

### 2.3. Changes in Ascorbic Acid Content During Low-Temperature Storage

**Figure 3:**
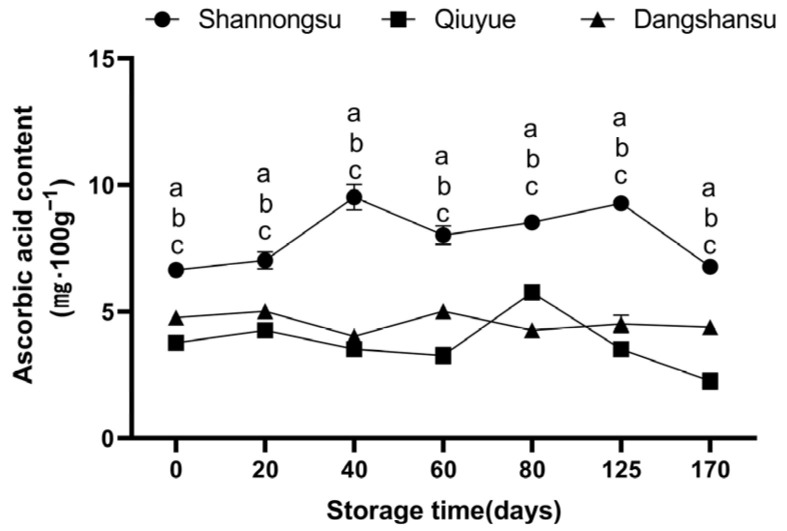
Changes in ascorbic acid content during storage. Values shown represent means ± SD (*n* = 3). Different letters indicate significant differences among varieties at the 0.05 level.

At 0 d after harvesting, the ascorbic acid content of the ‘Shannongsu’ pear was 6.65 mg·100 g^−1^, 1.39 and 1.77 times higher than that of the ‘Dangshansu’ and ‘Qiuyue’ pears, respectively (Figure 3). After 170 d of low-temperature storage, the ascorbic acid content of the ‘Shannongsu’ pear remained unchanged and was still significantly higher than that of the ‘Dangshansu’ and ‘Qiuyue’ pears.

### 2.4. Changes in the Content of Aromatic Substances During Low-Temperature Storage

**Figure 4:**
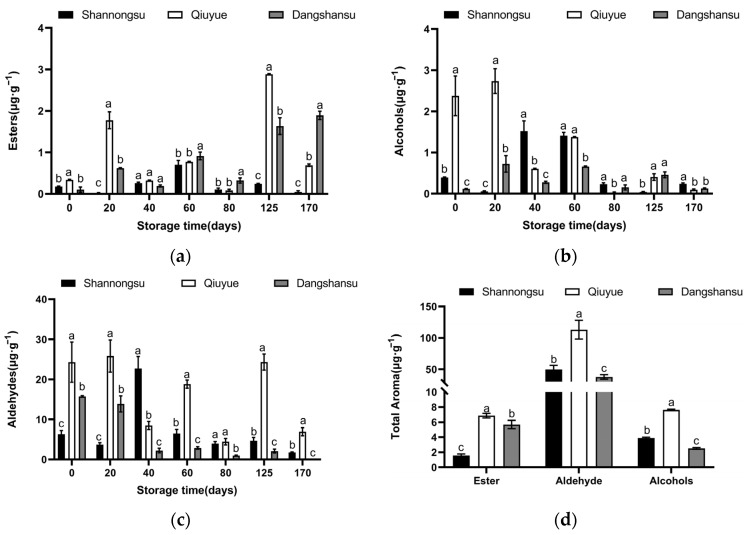
Changes in the contents of pear esters, alcohols, and aldehydes during storage: (**a**) changes in the content of esters; (**b**) changes in the content of alcohols; (**c**) changes in the content of aldehydes; (**d**) changes in total aroma contents. Values shown represent means ± SD (*n* = 3). Different letters indicate significant differences among varieties at the 0.05 level.

As shown in Figure 4a, during low-temperature storage, aromatic substances such as esters, aldehydes, and alcohols were present at low levels in the ‘Shannongsu’ pear. The highest value of esters was only 0.70 μg·g^−1^ at 60 days, much lower than 2.88 μg·g^−1^ in the ‘Qiuyue’ pear at 125 days. As shown in Figure 4d, the total level of esters released from ‘Shannongsu’ pear was 1.56 μg·g^−1^, which was 27% of the total esters released from the ‘Dangshansu’ pear and 23% of the total esters from the ‘Qiuyue’ pear. The total aldehyde and alcohol content released from the ‘Shannongsu’ pear during storage was slightly higher than that in the ‘Dangshansu’ pear. The total aldehydes content in ‘Shannongsu’ pears is 44% of that in ‘Qiuyue’ pears, while the total alcohols content is 51% of the ‘Qiuyue’ pears level.

### 2.5. Changes in the Expression of Key Genes Regulating Ethylene Synthesis During Low-Temperature Storage

**Figure 5:**
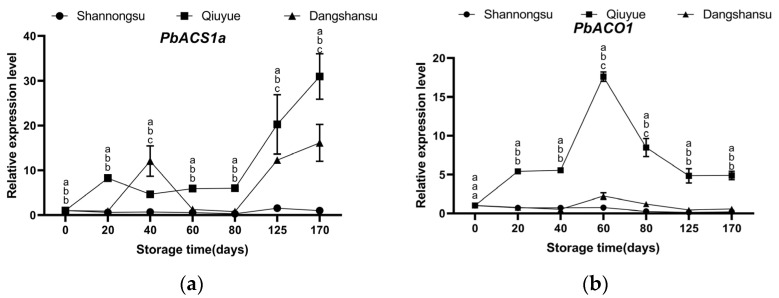
Changes in the relative expression levels of *PbACS1a* and *PbACO1* during storage: (**a**) Changes in the relative expression level of *PbACS1a*; (**b**) Changes in the relative expression level of *PbACO1*. Values shown represent means ± SD (*n* = 3). Different letters indicate significant differences among varieties at the 0.05 level.

**Figure A2:**
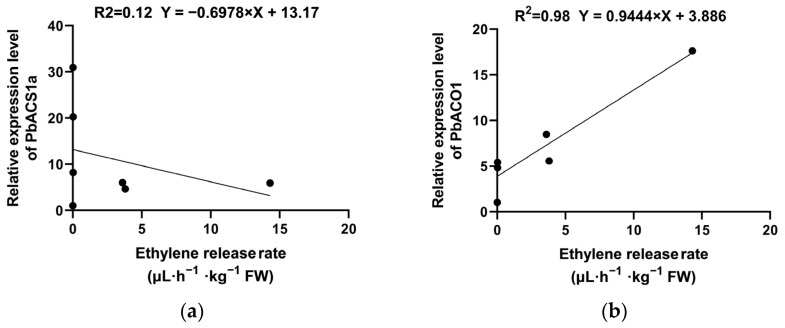
Correlation analysis of ethylene content and the expression levels of *PbACS1a* and *PbACO1* genes in ’Qiuyue’ pear: (**a**) Correlation analysis of ethylene content and the expression levels of *PbACS1a* gene in ’Qiuyue’ pear; (**b**) Correlation analysis of ethylene content and the expression levels of *PbACO1* gene in ’Qiuyue’ pear.

In ‘Shannongsu’ pear, the expressions of *PbACS1a* and *PbACO1* are maintained at relatively low levels, while the expression of *PbACO1* is significantly down-regulated. In contrast, in ‘Dangshansu’ pear and ‘Qiuyue’ pear, the expression of *PbACS1* shows an upward trend (Figure 5). In ‘Qiuyue’ pear, the change in the trend of the *PbACO1* expression level is significantly correlated with ethylene production (r = 0.99, Figure A2b).

### 2.6. Changes in the Expression of Texture-Related Genes During Storage

**Figure 6:**
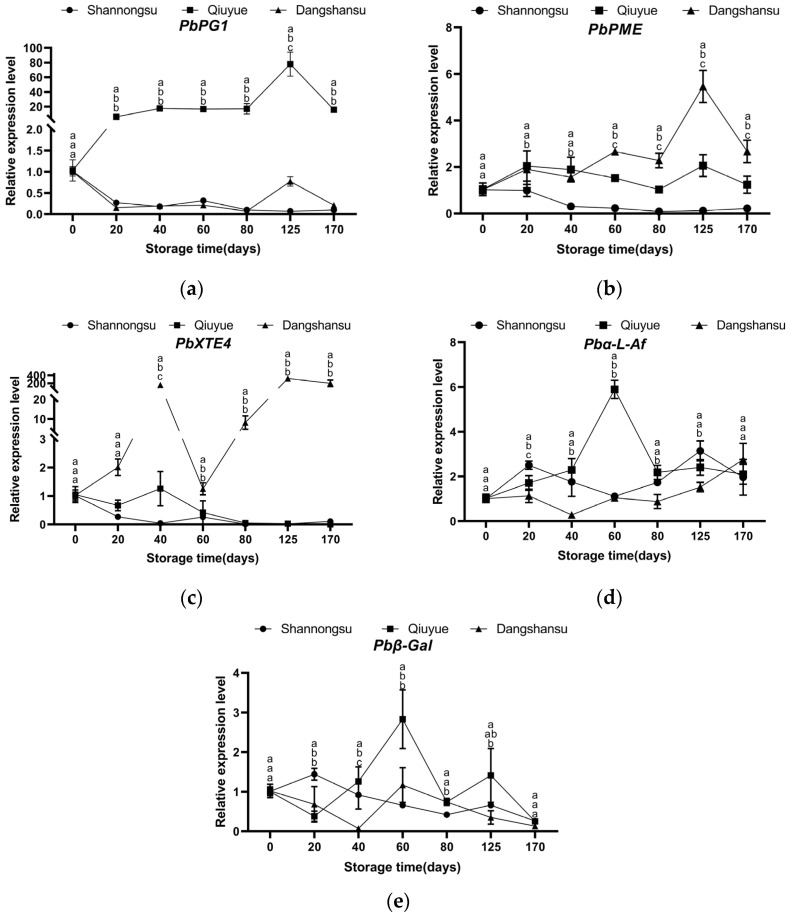
Changes in the relative expression levels of *PbPG1*, *PbPME*, *PbXET4*, *Pbα-L-Af*, *Pbβ-Gal* genes during storage: (**a**) Changes in the relative expression level of *PbPG1*; (**b**) Changes in the relative expression level of *PbPME*; (**c**) Changes in the relative expression level of *PbXET4*; (**d**) Changes in the relative expression level of *Pbα-L-Af*; (**e**) Changes in the relative expression level of *Pbβ-Gal*. Values shown represent means ± SD (*n* = 3). Different letters indicate significant differences among varieties at the 0.05 level.

As shown in Figure 6, during low-temperature storage, the expression levels of genes such as *Pbα-L-Af* and *Pbβ-Gal* were up-regulated in the ‘Qiuyue’ pear. In the ‘Dangshansu’ pear, the expression levels of genes such as *PbPG1*, *PbPME*, and *PbXET4* were up-regulated. However, in the ‘Shannongsu’ pear, the expression levels of *PbPG1*, *PbPME*, *PbXET4*, and *Pbβ-Gal* were always low, and no significant up-regulation was observed.

## 3. Discussion

During the fruit ripening process, endogenous ethylene enhances the permeability of the plasma membrane and accelerates plant respiration. It also promotes the transformation of organic substances in the pulp, ultimately leading to fruit ripening and softening [16,17]. Ethylene treatment significantly accelerates the decrease in firmness and increases the activity of cell wall hydrolases in ripe fruit [18]. The rate-limiting enzymes involved in ethylene biosynthesis are ACS and ACO. The ethylene yield and shelf life are closely related to the expression of ACS and ACO genes [19,20]. Apple fruits with silenced ACS1 expression exhibit very low levels of ethylene production, and their storage life at room temperature is significantly increased [21]. In this study, the ‘Shannongsu’ pear reached its peak ethylene release 60 days after low-temperature storage. This time point was later than that of the ‘Qiuyue’ pear. Moreover, at the peak, its ethylene release amount was relatively lower than that of the ‘Dangshansu’ pear. The difference in this ethylene release pattern might be one of the key factors contributing to the better storage tolerance of the ‘Shannongsu’ pear under low temperatures. Furthermore, the expression levels of the *PbACS1a* and *PbACO1* genes in the ‘Shannongsu’ pear were maintained at a relatively lower level compared with those in the ‘Dangshansu’ and ‘Qiuyue’ pears. Consequently, the weak ability of the ‘Shannongsu’ pear to synthesize ethylene is the key reason for the low ethylene release level and the slow decline in firmness. Additionally, low temperatures can further inhibit ethylene release and help maintain storage-related qualities.

Changes in the structure and composition of the cell wall, caused by multiple hydrolases, are directly responsible for fruit ripening and softening [22,23]. Up to thirty enzymes are involved in cell wall metabolism. However, PG, PME, β-Gal, and XET are generally considered closely associated with cell wall degradation as well as fruit ripening and softening [24,25,26,27,28,29]. Studies have shown that there is a positive correlation between the transcription level of *MdPG1* and the rate of fruit softening [27]. In this study, we found that during low-temperature storage, the expression of genes related to texture softening—namely *PbPG1*, *PbXET4*, and *PbPME*—was inhibited in the ‘Shannongsu’ pear. In contrast, the expression levels of these genes were up-regulated in the ‘Dangshansu’ pear and were much higher than those in the ‘Qiuyue’ and ‘Shannongsu’ pears. This may accelerate the degradation of its cell wall, resulting in a faster decrease in fruit hardness. The low expression levels of these genes related to fruit texture softening may be one of the reasons why this fruit is more resistant to storage conditions.

Volatile aroma compounds, as one of the important physiological indicators of fruit ripening, are closely related to ethylene regulation. An increase in ester content is closely associated with a decrease in fruit firmness, likely due to the accumulation of esters causing changes in the structure of the fruit cell wall, thereby accelerating the softening process. Conversely, alcohols and aldehydes may delay fruit softening by maintaining the stability and integrity of the fruit cell wall. Therefore, the composition and content of aroma compounds serve as critical indicators for evaluating fruit storability [30]. Research has shown that the poor storability of ‘Taishan Zaoxia’ apples is related to their high ester aroma content [31]. Other studies have also indicated that fruits with high ester content but low alcohol and aldehyde contents are less resistant to storage [6]. In this study, during low-temperature storage, the ester content in ‘Shannongsu’ pears remained consistently low, with a total ester release of only 1.56 μg·g^−1^, which was 27% and 23% of that in the ‘Dangshansu’ and ‘Qiuyue’ pears, respectively. This suggests that esters may be one of the factors affecting the storability of ‘Shannongsu’ pears.

Storability is a complex quantitative trait influenced by multiple factors. Traits such as fruit firmness, soluble solids, sugars, acids, and ascorbic acid content are crucial for evaluating fruit quality not only at the harvest stage but also during storage [21,32]. Studies have demonstrated that the firmness of kiwifruits from storable strains decreases more slowly, and fruit inclusions are depleted to a lesser extent in the later stages [33]. After fruit harvest, the titratable acid and ascorbic acid contents gradually decrease due to respiration. In this study, the sugar and acid fractions of the three pear varieties were measured on the day of harvesting. The sweetness values of the three pear varieties ranked from high to low were as follows: ‘Shannongsu’ pear > ‘Dangshansu’ pear > ‘Qiuyue’ pear (Appendix A). Furthermore, after 170 days of low-temperature storage, the ascorbic acid content, soluble solids, titratable acid, sugar–acid ratio, and aroma content of the ‘Shannongsu’ pear did not change significantly. As a result, this pear could still maintain good flavor and nutritional quality. Therefore, low-temperature storage can help maintain the freshness, flavor, and nutritional qualities of the ‘Shannongsu’ pear. During production, it can be used to extend the product’s supply period with lower preservation costs, enabling us to achieve a stable supply in the middle and high-end pear markets in China during the autumn, winter, and spring seasons.

‘Shannongsu’ pear is a new variety selected from a cross between the ‘Xinli No.7’ (‘Korla’ × ‘Zaosu’) and ‘Dangshansu’ pears [34]. In this study, the expression levels of *PbACO1* and *PbACS1a* as well as ethylene production in both ‘Shannongsu’ and ‘Dangshansu’ pears were relatively low. Additionally, the storage traits of the ‘Shannongsu’ pear were better than those of the ‘Dangshansu’ pear. Since the storage trait is a quantitative trait controlled by multiple genes, it is hypothesized that the storage-tolerance trait of ‘Shannongsu’ pear—a superlative trait resulting from the accumulation of multiple genes—may be inherited from the ‘Korla’ and ‘Dangshansu’ pears. Therefore, it is crucial to further explore the rich pear germplasm resources in China. This includes using the ‘Korla’ pear, ‘Dangshansu’ pear, and other traditional high-quality late-ripening varieties as parents to construct hybrid segregating populations. Studying and exploring the fruit formation and genetic mechanisms associated with fruit quality will contribute to breeding new high-quality late-ripening varieties in the future.

## 4. Materials and Methods

### 4.1. Materials

The experimental materials comprised ‘Shannongsu’, ‘Dangshansu’, and ‘Qiuyue’ pear cultivars harvested from Guanxian Breeding Base. Healthy fruits with uniform maturity, free from pests, diseases, and mechanical damage were selected (63 fruits per cultivar) and immediately transported to the cold storage facility at the College of Horticulture, Shandong Agricultural University. The storage temperature was set at 0 ± 0.5 °C. The humidity was between 90 and 95. Samples were collected on days 0, 20, 40, 60, 80, 125, and 170 after storage. At each time point, nine fruits per cultivar were randomly chosen to determine the firmness, ethylene release rate, and aroma components. After measurements, flesh tissues were sliced, rapidly frozen in liquid nitrogen, and stored at −80 °C.

### 4.2. Determination of Firmness

Firmness was determined using the method described by Zhang et al. [14]. A P/2 column probe (2-millimeter diameter) was used to perform whole fruit puncture with a TA.XT plus texturizer from Stable Microsystems, Godalming, UK. Pre-measurement speeds: 2 mm·s^−1^; speed in measurement: 1 mm·s^−1^; post-measurement speed: 5 mm·s^−1^; puncture depth: 10 mm; minimum sensing force: 10 g. At each time point, three fruits were randomly selected. Four points at the equatorial position of each fruit were selected for measurement. Texture Exponent 32 was used for the automatic analysis and calculation of values.

### 4.3. Determination of Ethylene Production

The ethylene production was measured using a Shimadzu GC-9A gas chromatograph. The chromatographic column was RTX-5MS from Agilent Technologies (Tokyo, Japan). Before measurement, the experimental material was sealed in a 2 L glass container and left to stand at room temperature for 12 h. The gas chromatograph conditions used were as follows: carrier gas H_2_ flow rate, 40 mL·min^−1^; carrier gas N_2_ flow rate, 25 mL·min^−1^; air flow rate, 400 mL·min^−1^; separation column temperature 70 °C; vaporization chamber and detector temperature, 120 °C. To extract the gas from the pears in the sealed glass container, a 1 mL syringe was used and the gas was injected into the sample inlet of the gas chromatograph. This extraction and injection process was repeated three times to obtain the average value [35].

### 4.4. Determination of Soluble Sugar Content

The determination was carried out by the anthraquinone colorimetric method [36,37]. The steps are as follows: Accurately weigh out 0.3 g of the fruit sample for testing, and place it in a 15-mL centrifuge tube. Add 10 mL of distilled water, seal the tube, and then label it. Boil the tube in a water bath for 30 min. Filter the solution into a 25-mL volumetric flask. Repeat the extraction process once and then adjust the volume to the mark. Clean and dry a glass tube. Successively add 0.1 mL of the extract, 1.9 mL of distilled water, 0.5 mL of anthrone ethyl acetate, and 5 mL of concentrated sulfuric acid into the tube. Seal the tube, shake well, and boil in water for 1 min. Then, allow it to cool. Use a Shimadzu UV-2450 UV–visible spectrophotometer to measure the absorbance at 630 nm. Set the blank to zero. Repeat the measurement three times.

### 4.5. Determination of Titratable Acid Content

The titration method was employed for determination [38]. Specifically, 3 g of the sample was weighed and then diluted to 50 milliliters with water. The mixture was allowed to stand at 4 °C for 35 min. After that, 10 mL of the supernatant was transferred into a conical flask, and 2 drops of 1% phenolphthalein indicator were added. Subsequently, titration was carried out using 0.02 mol·L^−1^ sodium hydroxide solution until a persistent pink color appeared in the solution and persisted for at least 30 s. The volume of sodium hydroxide solution consumed was recorded. For the blank test, an equal volume of distilled water was used to replace the filtrate. Each measurement was repeated three times.

### 4.6. Determination of Ascorbic Acid Content

The ascorbic acid content was quantified using the BC4630 AsA/T-AsA assay kit (Beijing Solarbio Science & Technology Co., Ltd., Beijing, China) according to the manufacturer’s protocol. This method utilizes the inherent reducing capacity of ascorbic acid to reduce Fe^3+^ to Fe^2+^, forming a pink complex with 2,2′-bipyridine that exhibits a distinct absorbance peak at 525 nm, enabling the quantitative determination of ascorbic acid in the sample.

### 4.7. Extraction and Detection of Sugar Components

High-performance liquid chromatography (HPLC) employs Filip’s method for sample preparation and analysis [39]. The sample solution was injected and the peak area was recorded. Then, the content of each sugar based on the standard curve was calculated. The sweetness calculation formula is as follows: 100 × glucose + 200 × fructose + 145 × sucrose [40].

### 4.8. Extraction and Detection of Aromatic Substances

The research methods of predecessors were adopted to extract and detect fruit aromatic substances [17,41]. A GC/MS-QP2010 (Shimadzu, Kyoto, Japan) was used to determine the relative content of the compounds. To begin, the extraction head was aged at 250 °C for 40 min. Next, 5 g of the sample was weighed for testing and placed into a 50 mL conical flask. A total of 5 μL of 3-nonanone (0.4 mg·mL^−1^) was added to the flask, a rotor was inserted, and the flask was sealed. After that, the flask was equilibrated at 45 °C for 5 min and extracted at 45 °C for 40 min. Three biological replicates were conducted for each sample. Identification of the compounds was achieved by comparison with the NIST 2017 library and determining the linear retention index. The relative content was further determined based on the total ion chromatogram and the internal standard peak area.

### 4.9. Quantitative Real-Time Polymerase Chain Reaction (qRT-PCR)

The RNAprep Pure Polysaccharide Polyphenol Plant Total RNA extraction kit (Tiangen, Beijing, China, DP441) was employed. Quantitative real-time PCR (qPCR) was performed using Tip Green qPCR SuperMix (Quanshijin, Beijing, China, AQ321-02). The PbActin gene was used as an internal control. All primers were designed with Primer Premier 6 and synthesized by Shenggong Bioengineering (Shanghai) Company Limited (Shanghai, China); the primer sequences are presented in Table A2.

### 4.10. Statistical Analysis

All data are presented as the mean ± standard deviation (SD) of three independent replicates. Data analysis was carried out using SPSS 21.0. A one-way analysis of variance (ANOVA) was conducted to compare the means among different groups. Multiple comparisons were conducted using Tukey’s Honest Significant Difference (Tukey HSD) test. A *p*-value < 0.05 was considered statistically significant.

## 5. Conclusions

Low temperature may primarily maintain the good fresh consumption quality of ‘Shannongsu’ pears by inhibiting the expression of genes related to ethylene and ester synthesis as well as cell-wall-encoding genes such as PG1. Therefore, in production, a longer marketing period of ‘Shannongsu’ pears can be maintained through low-temperature storage.

## Figures and Tables

**Figure A1:**
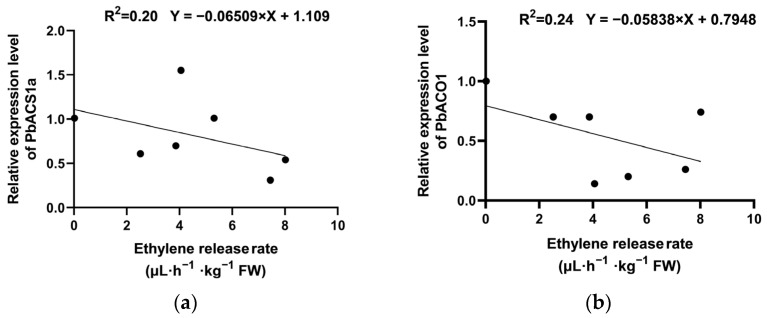
Correlation analysis of ethylene content and the expression levels of *PbACS1a* and *PbACO1* genes in ’Shannongsu’ pear: (**a**) Correlation analysis of ethylene content and the expression levels of *PbACS1a* gene in ’Shannongsu’ pear. (**b**) Correlation analysis of ethylene content and the expression levels of *PbACO1* gene in ’Shannongsu’ pear.

**Figure A3:**
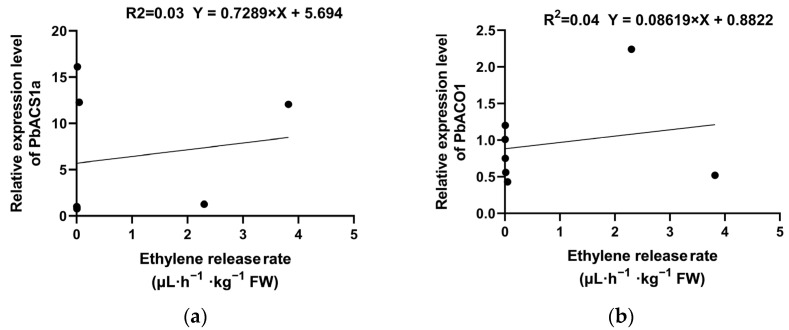
Correlation analysis of ethylene content and the expression levels of *PbACS1a* and *PbACO1* genes in ’Dangshansu’ pear: (**a**) Correlation analysis of ethylene content and the expression levels of *PbACS1a* gene in ’Dangshansu’ pear; (**b**) Correlation analysis of ethylene content and the expression levels of *PbACO1* gene in ’Dangshansu’ pear.

**Figure A4:**
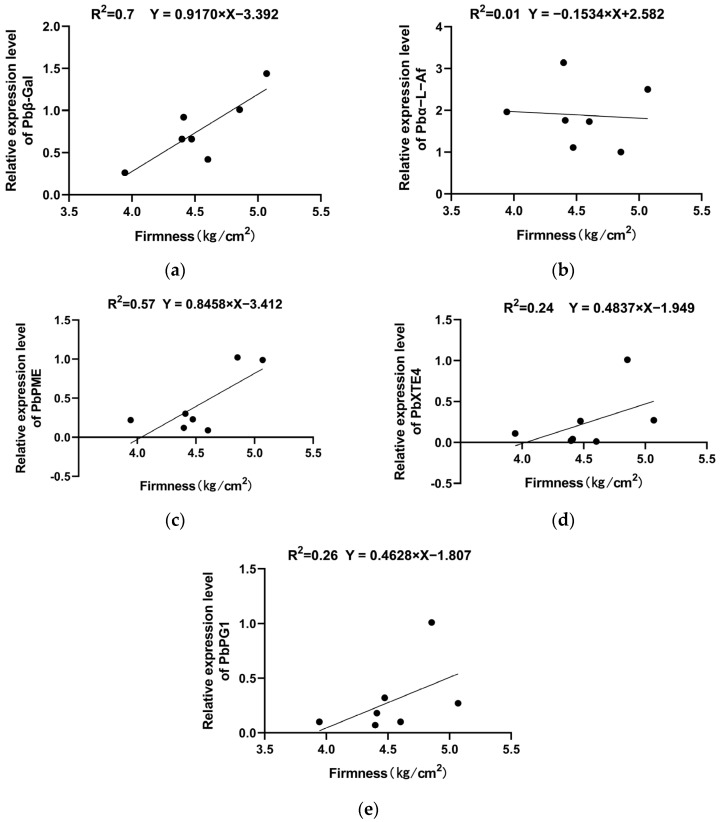
Correlation analysis between the firmness of ‘Shannongsu’ pear and the expression levels of *PbPG1*, *PbPME*, *PbXET4*, *Pbα-L-Af*, and *Pbβ-Gal* genes: (**a**) Correlation analysis between the firmness of ‘Shannongsu’ pear and the expression levels of *Pbβ-Gal* gene. (**b**) Correlation analysis between the firmness of ‘Shannongsu’ pear and the expression levels of *Pbα-L-Af* gene. (**c**) Correlation analysis between the firmness of ‘Shannongsu’ pear and the expression levels of *PbPME* gene. (**d**) Correlation analysis between the firmness of ‘Shannongsu’ pear and the expression levels of *PbXET4* gene. (**e**) Correlation analysis between the firmness of ‘Shannongsu’ pear and the expression levels of *PbPG1* gene.

**Figure A5:**
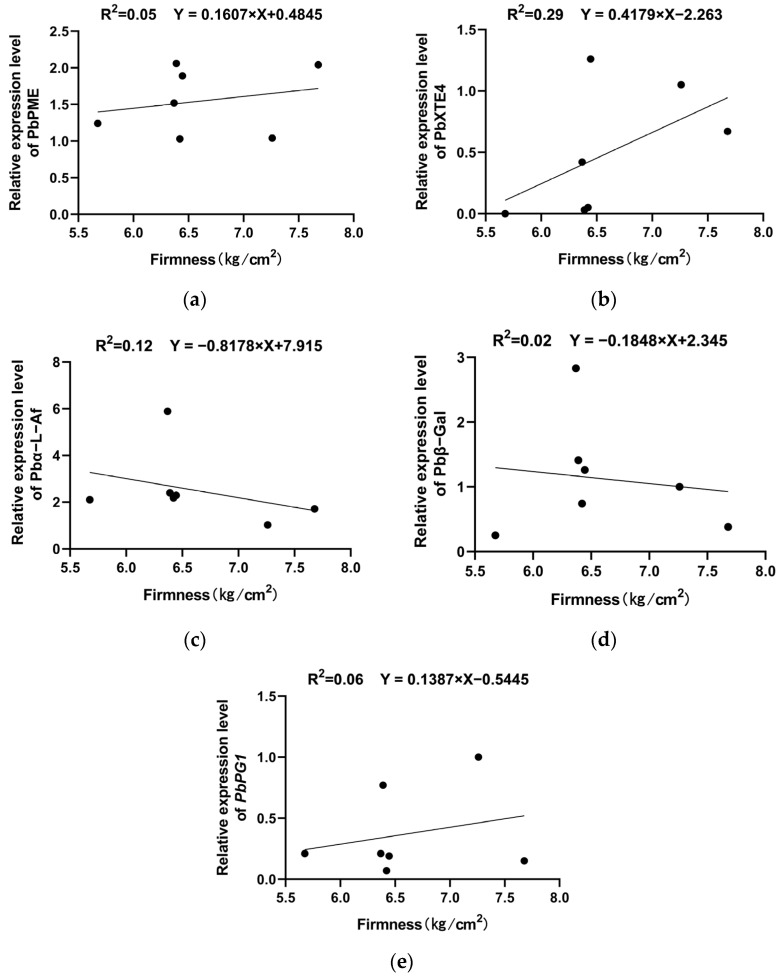
Correlation analysis between the firmness of ‘Qiuyue’ pear and the expression levels of *PbPG1*, *PbPME*, *PbXET4*, *Pbα-L-Af*, and *Pbβ-Gal* genes: (**a**) Correlation analysis between the firmness of ‘Qiuyue’ pear and the expression levels of *PbPME* gene. (**b**) Correlation analysis between the firmness of ‘Qiuyue’ pear and the expression levels of *PbXET4* gene. (**c**) Correlation analysis between the firmness of ‘Qiuyue’ pear and the expression levels of *Pbα-L-Af* gene. (**d**) Correlation analysis between the firmness of ‘Qiuyue’ pear and the expression levels of *Pbβ-Gal* gene. (**e**) Correlation analysis between the firmness of ‘Qiuyue’ pear and the expression levels of *PbPG1* gene.

**Figure A6:**
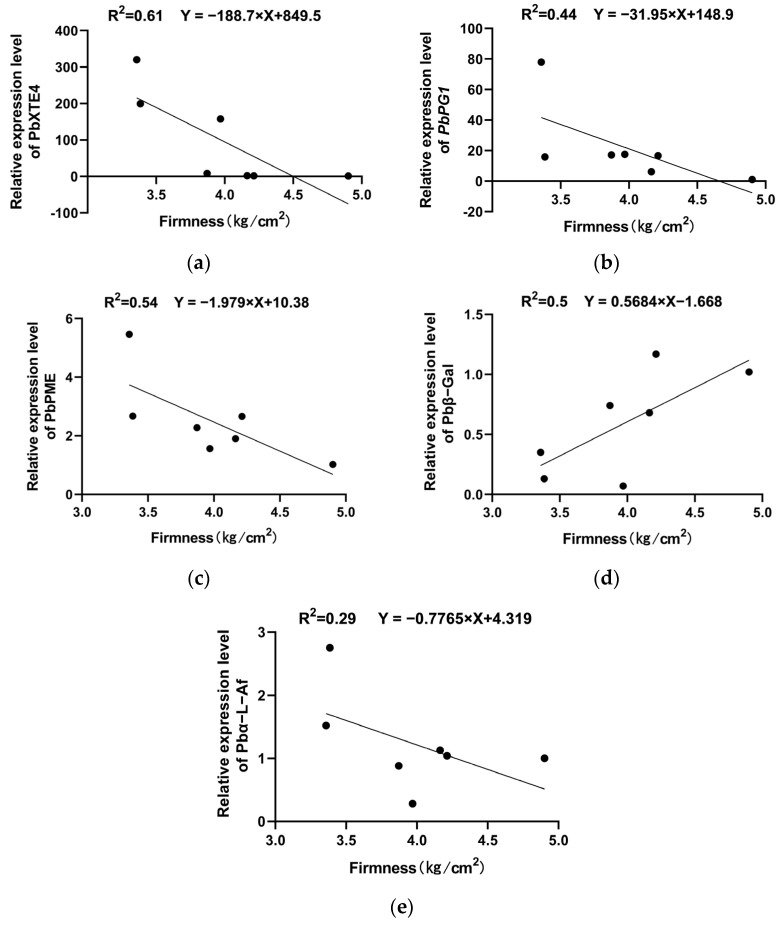
Correlation analysis between the firmness of ‘Dangshansu’ pear and the expression levels of *PbPG1*, *PbPME*, *PbXET4*, *Pbα-L-Af*, and *Pbβ-Gal* genes: (**a**) Correlation analysis between the firmness of ‘Dangshansu’ pear and the expression levels of *PbXET4* gene. (**b**) Correlation analysis between the firmness of ‘Dangshansu’ pear and the expression levels of *PbPG1* gene. (**c**) Correlation analysis between the firmness of ‘Dangshansu’ pear and the expression levels of *PbPME* gene. (**d**) Correlation analysis between the firmness of ‘Dangshansu’ pear and the expression levels of *Pbβ-Gal* gene. (**e**) Correlation analysis between the firmness of ‘Dangshansu’ pear and the expression levels of *Pbα-L-Af* gene.

## Data Availability

The original contributions presented in this study are included in the article. Further inquiries can be directed to the corresponding authors.

## Appendix A

**Table A1:** The sugar and acid fractions of the three pear cultivars. Values shown represent means ± SD (*n* = 3). Different letters indicate significant differences among varieties at the 0.05 level.

Varieties	Sucrose (mg·mL^−1^)	Glucose (mg·mL^−1^)	Fructose (mg·mL^−1^)	Sorbitol (mg·mL^−1^)	Total Sugar (mg·mL^−1^)	Sweetness (mg·mL^−1^)
Shannongsu	1.68 ± 0.04 b	2.26 ± 0.04 b	4.22 ± 0.08 a	2.46 ± 0.05 a	10.61 ± 0.21 a	11.62 ± 0.23 a
Dangshansu	0.15 ± 0.01 c	2.68 ± 0.03 a	4.26 ± 0.05 a	2.09 ± 0.01 b	9.18 ± 0.09 b	10.31 ± 0.12 b
Qiuyue	3.51 ± 0.04 a	1.14 ± 0.01 c	3.12 ± 0.02 b	1.26 ± 0.07 c	9.04 ± 0.03 b	10.28 ± 0.08 b

**Table A2:** Primers of softening-related enzyme genes in pear fruit for the qPCR amplification.

Gene Name	Primer Sequence (5′–3′)	
*PbACS1a*	CAAGAACAAGCACAAGTGA	ATTGGTGAATGAGGAGACA
*PbACO1*	ACCACTCCATTGTCATAAAC	GGTTGTAGAACGAGGCTAT
*PbPG1*	GTTGTGGTCATTTGTTGTTG	CGGACACCCTATCATCAA
*PbPME*	GATGGCTTGAGTGGAATG	GCAACAGTGAACTTACCG
*PbXET4*	TCCATTCCTACTCCATCTTC	AATCTGTCTTGACCCTACC
*Pbβ-Gal*	TGGTCCATCAGCATTCTT	TGTAGTTGTATCGGTCTCAT
*Pbα-L-Af*	GCCCTTTGACTTGAGATATG	ATCCAACTGACGAGAAGAA
